# Metagenomics reveals functional synergy and novel polysaccharide utilization loci in the *Castor canadensis* fecal microbiome

**DOI:** 10.1038/s41396-018-0215-9

**Published:** 2018-07-16

**Authors:** Zachary Armstrong, Keith Mewis, Feng Liu, Connor Morgan-Lang, Melanie Scofield, Evan Durno, Hong Ming Chen, Kevin Mehr, Stephen G. Withers, Steven J. Hallam

**Affiliations:** 1 Genome Science and Technology Program, University of British Columbia, 2329 West Mall, Vancouver, BC V6T 1Z4 Canada; 2 Department of Chemistry, University of British Columbia, 2036 Main Mall, Vancouver, V6T 1Z1 BC Canada; 3 Department of Microbiology & Immunology, University of British Columbia, Vancouver, V6T 1Z1 BC Canada; 4 Centre for High-Throughput Biology, University of British Columbia, Vancouver, V6T 1Z3 BC Canada; 5 Department of Biochemistry and Molecular Biology, University of British Columbia, Vancouver, V6T 1Z3 BC Canada; 6 ECOSCOPE Training Program, University of British Columbia, Vancouver, V6T 1Z3 BC Canada; 7 Graduate Program in Bioinformatics, University of British Columbia, Vancouver, V6T 1Z4 BC Canada; 8 Peter Wall Institute for Advanced Studies, University of British Columbia, Vancouver, V6T 1Z1 Canada

## Abstract

The North American beaver (*Castor canadensis*) has long been considered an engineering marvel, transforming landscapes and shaping biological diversity through its dam building behavior. While the beaver possesses conspicuous morphological features uniquely adapted for the use of woody plants as construction materials and dietary staples, relatively little is known about the specialized microorganisms inhabiting the beaver gastrointestinal tract and their functional roles in determining host nutrition. Here we use a combination of shotgun metagenomics, functional screening and carbohydrate biochemistry to chart the community structure and metabolic power of the beaver fecal microbiome. We relate this information to the metabolic capacity of other wood feeding and hindgut fermenting organisms and profile the functional repertoire of glycoside hydrolase (GH) families distributed among and between population genome bins. Metagenomic screening revealed novel mechanisms of xylan oligomer degradation involving GH43 enzymes from uncharacterized subfamilies and divergent polysaccharide utilization loci, indicating the potential for synergistic biomass deconstruction. Together, these results open a functional metagenomic window on less conspicuous adaptations enabling the beaver microbiome to efficiently convert woody plants into host nutrition and point toward rational design of enhanced enzyme mixtures for biorefining process streams.

## Introduction

Microbial communities inhabiting the mammalian digestive tract, gut microbiomes, affect host health and mediate essential services including dietary access to recalcitrant glycans such as starches and fibers [1]. With respect to digestion, the taxonomic composition of these communities correlates with host diet and nutrient acquisition strategies across different mammalian lineages [2]. Multiple studies indicate that mammalian gut microbiomes consist of specialized communities that respond to complex glycans derived from specific dietary sources such as lignocellulosic biomass and release products that can be absorbed into the digestive tract [2–6]. Consistent with these observations, the digestive tracts of herbivores and wood-feeding (xylotrophic) organisms harbor microbial communities enriched in genes or gene cassettes encoding the corresponding biocatalysts and polysaccharide utilization systems [7–12]. These communities provide a frame of reference for understanding how lignocellulosic biomass is converted into dietary macronutrients, as well as a deep reservoir of genomic information with potential biotechnological applications [13].

The North American beaver, *Castor canadensis*, provides a useful animal model for the study of xylotrophic microbiomes, as its diet is largely composed of bark, shoots, leaves, and other fibers from hardwood deciduous trees such as poplar, aspen, and cottonwood, which have commercial value in the forestry sector [14, 15]. Hardwoods such as poplar typically have a total polysaccharide content comprising 60–80% of the dry mass of the wood [16]. Some 35–50% of this dry mass consists of cellulose followed by hemicellulose (20% primarily glucuronoxylan) and pectin. Previous studies have shown that beavers are capable of digesting up to 32% of the available cellulose in consumed hardwoods [17]. However little is known about the utilization of the hemicellulose component by the beaver microbiome, and much less about the enzyme repertoire effecting its deconstruction.

Gruninger and colleagues recently used small subunit ribosomal RNA (SSU rRNA) gene sequencing to profile the microbial community structure of beaver cecum and feces, indicating a typical mammalian hindgut community dominated by Bacteroidetes and Firmicutes [18]. As a hindgut fermenter, commensal microbes in the lower digestive tract of the beaver are expected to mediate the degradation and fermentation of complex sugars to provide short chain fatty acids that provision host nutrition [19]. Given that the proclivity of beavers to consume wood differentiates them from other hindgut fermenting herbivores, several questions arise from the initial microbiome study: At what taxonomic level does the beaver microbiome differ from other xylotrophic and hindgut fermenting organisms? Does the microbiome encode specialized genes or gene cassettes mediating complete conversion of lignocellulosic biomass? Are some components of the lignocellulose targeted for digestion more than others? To what extent are these functions distributed at the population and community levels? Could analysis of these differences reveal new insight into sequential biomass deconstruction of wood-based fibers transferrable to industrial process streams? To begin answering these questions, we used a combination of SSU rRNA gene sequencing, shotgun metagenomics and functional screening to evaluate the community structure and metabolic potential of the beaver microbiome in relation to other wood-feeding organisms and to recover activities mediating lignocellulosic biomass deconstruction.

## Results

**Fig. 1:**
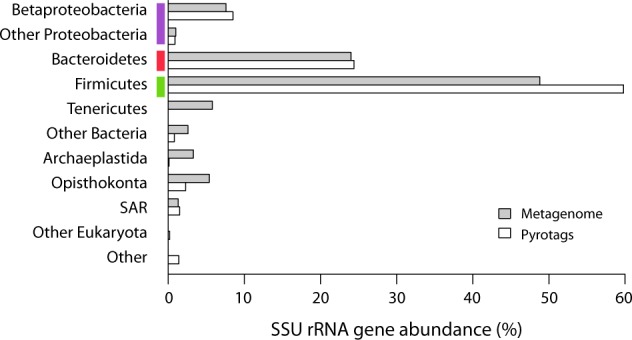
Beaver fecal community composition. The relative abundance of 16s rDNA genes found in the metagenome are compared to those identified by pyrotags. Both methods reveal a metagenome dominated by Firmicutes (green), Bacteroidetes (red) and Proteobacteria (purple) phyla

To profile the microbial community composition of the beaver fecal microbiome, we performed 454 pyrotag sequencing of the V6-V8 region of the SSU rRNA gene with three-domain resolution on composite fecal samples from 2 captive beavers. A total of 12,579 rRNA pyrotag sequences, recovered from the fecal sample, were clustered with a 97% similarity cut-off into 404 operational taxonomic units (OTUs) after singleton removal. Of the OTUs identified, only two could not be affiliated with described microbial taxa based on homology to sequences in the Silva database [20] (Fig. 1). One of these OTUs showed 99% identity to the *Castor canadensis* mitochondrial DNA sequence [21], while the other had at most 75% sequence identity to SSU rRNA sequences from uncultured bacteria. The majority of sequences were affiliated with the bacterial phyla Firmicutes (214 OTUs, 58.4%) and Bacteroidetes (93 OTUs, 24.4%). Within the Firmicutes, 200 OTUs were affiliated with the class Clostridia (55.9%), with 143 OTUs (43.6%) affiliated with the family Lachnospiraceae, which is known to harbor xylanotrophic, butyric acid producing members [22]. Within the Bacteroidetes, 68 of the 93 OTUs (21.3%) were affiliated with the class Bacteroidia, with 39 OTUs (15.5%) affiliated with the uncultivated S24-7 group. In a recent cultivation-dependent study, S24-7 comprised approximately 4% of the beaver fecal microbiome prior to methanogenic enrichment on different lignocellulosic biomass substrates [23]. Overall, these results are consistent with the observations of Gruninger and colleagues, although the proportions of Firmicutes and Bacteroidetes were not identical [18]. Additionally, the dominance of Firmicutes and Bacteroidetes is also seen in other wild rodents [24, 25]. As a high proportion of identified OTUs lacked cultured representatives (354 of 370 bacterial OTUs) specific metabolic roles could not be inferred with confidence. To this end we used shotgun metagenome sequencing to predict metabolic functions encoded in the beaver microbiome.

Shotgun metagenomic sequencing was conducted on the 454 platform from the same fecal DNA preparations used in SSU rRNA gene pyrotag analysis resulting in the production of 469.2 million base pairs (Mbp) of total sequence information (616,811 reads with average length 761 bp). Raw sequences were trimmed to Q30 quality score using prinseq lite+ [26] and assembled using MIRA [27], resulting in 75,523 contigs with an N50 of 1,787 bp and 130.5 Mbp of consensus sequence. One of these assembled contigs (beaver_rep_c845) had 99% identity with the 16 kb beaver mitochondria assembled by Horn and colleagues providing an additional reference for host population studies [21].

To explore potential bias in community structure based on pyrotag analysis, we examined SSU rRNA gene sequences recovered from the metagenome by comparing unassembled reads to the Silva SSU database using MetaPathways [28]. Of the unassembled reads 1,812 were annotated as having SSU rRNA genes (Supplementary Table 1). The majority of these sequences were affiliated with either Firmicutes (890) or Bacteriodetes (438), consistent with pyrotag results (Fig. 1). A notable exception was the relative abundance of Tenericutes within the metagenome, which was greater than that seen in the pyrotag data (percentages of 5.8% and 0.02% respectively). This may be due to amplification bias, as has previously been observed for Mycoplasma, the dominant Tenericutes genus identified in the beaver fecal metagenome [29]. In addition to bacteria, we detected SSU rRNA gene sequences affiliated with Archaeplastida (predominant class Liliopsida) and Opisthokonta (predominant class Insecta) at low abundance. The presence of these eukaryotic taxa in the beaver microbiome could reflect captive dietary intake or colonization post defecation.

Given the similarities between the taxonomic composition of the beaver fecal microbiome and other hindgut fermenters we initially looked for differences in carbohydrate active enzymes (CAZymes). Unassembled reads from the metagenome were queried against the CAZy database [30] using MetaPathways [28] and compared to 37 other mammalian species [2, 31, 32]. Because older datasets tended to have reduced and variable coverage per sample, unassembled metagenomic reads were compared using a variance stabilizing transformation (VST) [33]. CAZymes (all classes) constituted 3.85% of predicted genes within the beaver fecal microbiome compared to 2.89–6.95% for other organisms (Supplementary Table 2). Consistent with previous reports, CAZymes formed distinct clusters with glycoside hydrolase (GH) families encoding cellulases, hemicellulases, and pectinases overrepresented in herbivores, and families encoding glycosaminoglycanases overrepresented in carnivores [2, 5] (Supplementary Fig. 1). Overall, the beaver CAZyme profile was most similar to those of hindgut-fermenting herbivores.

To determine the metabolic context of GH families in the beaver fecal microbiome we reconstructed metabolic pathways on open reading frames (ORFs) predicted on assembled contigs and annotated using MetaPathways [28]. Of the 48,959 ORFs mapped to KEGG pathways, 9514 or 19.4% were associated with carbohydrate metabolism (Supplementary Table 3). In particular, ORFs encoding beta-glucosidase (*bglX*: K05349) and beta-galactosidase (*lacZ*: K01190), were similar in abundance to those of other mammals (*bglX* mean = 0.313 ± 0.14%, beaver = 0.347%; *lacZ* mean = 0.342 ± 0.13%, beaver = 0.368%).

Based on observed functional similarities between CAZymes and KEGG pathways present in the beaver fecal microbiome and other hindgut fermenting herbivores, we wondered if functional organization of these genes at the population or community levels could in part explain the capacity of the beaver to digest wood fibers efficiently. Contigs and functionally identified fosmids (described below) were binned into population genomes using MaxBin 2.0 [34] which were refined using the Anvi’o metagenomics workflow [35]. This binning produced 8 medium-quality (completeness >50% and contamination <10%) and 22 low-quality metagenome-assembled genomes (MAGs) [36]. Together these bins accounted for 6.2% of the assembled metagenome (Supplementary Table 4). Further analysis was confined to the medium-quality bins. We then used PhyloSift to assign taxonomy to these bins [37]. Two bins were assigned to the Bacteroidetes phylum (Bins 1 and 2), five to the order Clostridiales (3, 5, 7, 8, 13 and 16) and one to an undefined genus within the Burkholderiales order (Bin 6). Three Clostridiales bins could be further classified beyond the order level, with one classified at the Genus level as a *Flavobacterium* (Bin8), and two classified at the species level as *Coprococcus sp*. ART55/1 (Bin13) and *Eubacterium eligens* (Bin16). This species-level assignment was cross-validated using average nucleotide identity (ANI) to known isolate genomes, revealing that Bin13 had 97.5% ANI to the *Coprococcus sp*. ART55/1 genome, while Bin16 had 95.6% ANI to *Eubacterium eligens* strain ATCC 27750. The strain *Coprococcus sp*. ART55/1, which belongs to the family Lachnospiraceae, is likely involved in the generation of fermentation products within the beaver gut as it has previously been shown to produce butyrate [38].

The abundance of plant carbohydrate active GH families within the identified population genome bins was then investigated (Supplementary Table 5). All medium-quality bins exhibited incomplete enzyme repertoires involved in lignocellulosic biomass deconstruction consistent with combinatorial substrate conversion processes. One set of bins (1, 13, 16) contained a wide range of genes encoding hemicellulose degrading enzymes (average 2.1% of all ORFs), but lacked genes encoding enzymes necessary for glucuronoxylan debranching (GH families 67 and 115). Cellulose degradation appeared to be chiefly performed by bins 1, 2, 7, and 13, as these were the only bins to contain GH families with endo-cellulase activity. Three of the eight bins appeared to target pectins (1, 7, and 16) with Bin16, the *Eubacterium eligens* bin, having the most extensive array of pectin targeting genes. This fits well with the previous observations of pectin degradation by this species [39]. The *Coprococcus* bin (Bin13) contained plant biomass targeting GH and CE families (GH43, GH95, CE4) that were absent in the *Coprococcus sp*. ART55/1 genome, suggesting expanded ability to degrade carbohydrates by the strain found in the beaver feces. The remaining bins (2, 3, and 8) appeared to have a comparatively reduced capacity to degrade lignocellulosic biomass, but contained multiple enzymes for starch or glycogen degradation as well as simple glucosides and gluco-oligosaccharides present within the beaver diet or liberated by the endo-acting enzymes of co-occurring microorganisms.

While this analysis provided more specific information regarding metabolic potential of observed community members, specific CAZyme functions were inferred solely on the basis of sequence homology. Although enzyme activity often correlates with CAZy family, new activities are often identified within existing families, such as those identified by Ndeh et al. [40]. To better validate gene models we conducted functional screening to recover active CAZymes from large insert (fosmid) clones.

A fosmid library containing over 4,500 clones was constructed from the same DNA used in shotgun metagenome sequencing using the pCC1 copy control system expressed in *E. coli* EPI300. Activity assays were based on methods described by Mewis and colleagues [41, 42] but instead of chromogenic substrates we utilized cellobioside, xyloside, and xylobioside fluorogenic substrates bearing the 6-chloro-7-hydroxy-4-methylcoumarin aglycone, resulting in greater sensitivity with improved signal to noise ratio [43]. We combined the three substrates in a multiplex format to reduce both the time required and materials costs (Supplementary Fig. 2). Multiplex screening identified 51 fosmids that hydrolyzed at least one of the three fluorogenic substrates (z-score > 3); a hit rate of 1 in 88 (Supplementary Fig. 3). Substrate specificities of active clones recovered in multiplex screening were further assessed against a panel of nine separate fluorogenic substrates (Supplementary Fig. 4). A majority of clones were most active against either β-glucosides or β-xylosides However, six clones displayed higher activities against alternative substrates including arabinose (06_E19, 09_O03, 09_O15), galactose (10_J12), lactose (9_I18), and mannose (5_B01). This suggests that either the active enzymes encoded on these fosmids possess broad substrate specificities, or that multiple functions are encoded and expressed from individual clones consistent with gene cassettes e.g., cellulosomes or polysaccharide utilization loci (PULs) involved in the extracellular deconstruction of insoluble biomass [44] and the utilization of soluble carbohydrates within the cell respectively [45].

**Fig. 2:**
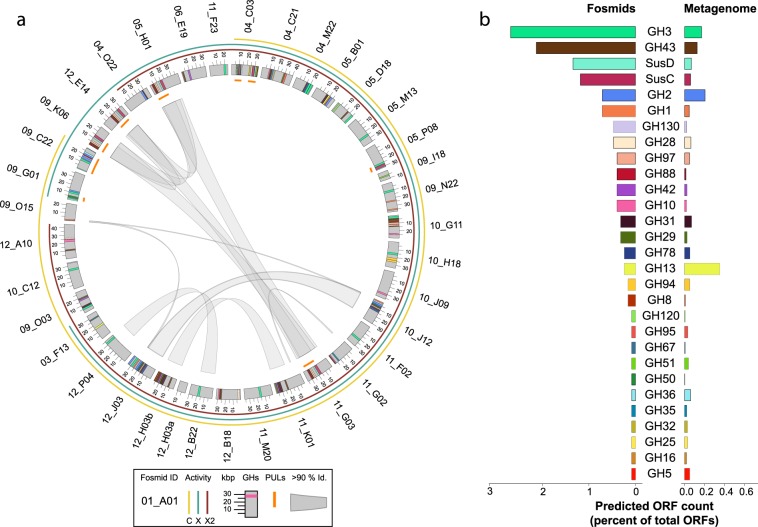
Fosmids identified from high throughput screening. **a** Schematic representing the identified fosmids, gene presence and similarity. Gray bars represent each fosmid and are proportional to their length. Fosmids sharing 100% identity with another fosmid were removed as duplicates. Connections in the center represent areas of 90% or greater nucleotide identity between fosmids as identified by BLASTN. Inner track represents the locations of identified PULs. Outer colored track represents activities identified for each fosmid from functional screening (C: CMU-cellobiose, X: CMU-xylose, X2: CMU-xylobiose). Colored bars within each fosmid represent GH domains as predicted by BLASTP against the CAZy database. **b** Histogram displays color encoding of GH gene families as well as abundance of each family in the complete fosmid dataset compared to the abundance of the same gene families in the unassembled metagenomic dataset. The abundance relative to the total number of ORFs is shown in parentheses

To identify individual genes or gene cassettes mediating substrate conversion we fully sequenced the 51 active clones. Reads were assembled using ABySS [46] and ORFs predicted and annotated using the MetaPathways pipeline [28]. Comparison between sequences identified 38 non-redundant clones based on >95% similarity across >90% of insert length (Fig. 2a). The reads mapped per kilobase of fosmid per million mapped reads (RPKM) from the metagenome was highly variable between fosmids, with an average value of 8.7 ± 13.0 per fosmid; a maximum of 81.9 and a minimum of 0.1 (Supplementary Table 6). Few fosmids had greater than 90% metagenome coverage (04_O22, 05_H01, 12_E14) with two fosmids (09_N21, 09_N22) recruiting less than 5% of their total sequence length (Supplementary Table 6). LCA assignment of 1,267 predicted ORFs indicated that 13 active clones were affiliated with Firmicutes and 38 were affiliated with Bacteroidetes donor genotypes (Supplementary Fig. 5). When queried against the medium-quality metagenomic bins, 5 active clones had >97% identity to a contig larger than 1 kb (Supplementary Table 6). This number includes 04_O22, the only fosmid to be assigned to a medium-quality bin. The four remaining fosmids (05_H01, 09_K06, 11_G03, and 12_E14) all aligned with over 75% of their lengths to Bin 1.

Queries of sequenced fosmids against the CAZy database identified 135 GH genes from 28 GH families encompassing 11.13% of the annotated ORFs (Fig. 2b). Unexpectedly, active fosmids harbored only 5 GH genes from families with annotated cellobiohydrolases or endoglucanases, one each from GH families 5 and 51, and three from GH8 with none from the cellulolytic GH families 6, 7, 9, 12, 26, 44, 45, 48, 74, or 124. As the metagenome encodes for a total of 1,085 cellulases from 9 of 14 cellulase families (0.15% of all predicted metagenome ORFs) it may be that we have only captured the most abundant taxa within the fosmid library and that cellulose is being degraded by rarer taxa within the community. In contrast, there was an abundance of genes encoding hemicellulose converting enzymes, particularly xylan. The most abundant GH family recovered was GH3, which contains both β-xylosidases and β-glucosidases. This was followed in abundance by GH43, a family containing both β-xylosidases and xylanases [47]. Within the unassembled metagenome, 115 GH43 genes (0.015% of all metagenomic genes) from 23 subfamilies were identified, the second highest diversity of GH43 subfamilies seen in any of the mammalian metagenomes analyzed (Supplementary Table 7). A total of 27 GH43 genes (1.8% of fosmid genes) from 10 subfamilies were identified on active clones (Table 1). Three GH43 genes, 12_H03 (two genes - subfamilies 2 and 7) and 12_J03 (subfamily 28), belonged to subfamilies containing no previously characterized members thus were of unknown specificities. Genes encoding xylan side-chain removing enzymes (α-glucuronidase from GH67; α-galactosidases from GH 28 and 97) were also present. Interestingly, we identified a number of genes encoding multiple GH domains (Supplementary Fig. 6). Four of these encoded predicted endo-acting and exo-acting domains. These include 4_C21-10 and 12_B18-19 which contain both GH43 and GH10 domains and are likely involved in xylan degradation, as well as 10_G11-03 and 12_H03-13, both of which contain GH43 and GH8 domains consistent with a role in xyloglucan or xylan degradation. The presence of both endo- and exo-glycosidase domains within the same protein can lead to synergism in efficient deconstruction of polysaccharides, as has been observed previously [48].

**Table 1:** GH43 subfamilies identified on functionally active fosmids

Fosmid	ORF	GH43 subfamily
Beaver_04_C21	10	12
Beaver_04_C21	11	1
Beaver_04_M22	11	24
Beaver_04_O22	25	19
Beaver_05_D18	17	11
Beaver_05_H01	3	10
Beaver_06_E19	1	11
Beaver_09_K06	1	10
Beaver_10_G11	3	2
Beaver_10_G11	4	29
Beaver_10_G11	8	29
Beaver_10_J12	4	28
Beaver_10_J12	7	24
Beaver_11_G02	25	1
Beaver_11_G03	16	10
Beaver_11_K01	12	12
Beaver_11_K01	19	29
Beaver_12_A10	9	12
Beaver_12_B18	18	11
Beaver_12_B18	19	12
Beaver_12_E14	11	10
Beaver_12_H03	10	29
Beaver_12_H03	12	7
Beaver_12_H03	13	2
Beaver_12_H03	3	12
Beaver_12_J03	15	24
Beaver_12_J03	18	28

To better understand the substrate specificities and activities of the enzymes present, we focused our attention on the uncharacterized GH43 subfamilies identified by functional screening. To this end we generated constructs that were used to overexpress and purify recombinant proteins (12_H03-13 from subfamily 2, 12_H03-12 from subfamily 7, and 12_J03-18 from subfamily 28). Since 12_H03-13, also contained a GH8 domain, we created two additional constructs in which the GH8 and GH43 domains were inactivated independently by mutation of the catalytic acid residue (GH8 domain mutant H03-13_E507A and GH43 domain mutant H03-13_E209A). Of the three wild-type enzymes, two had detectable cleavage activity on monosaccharide aryl glycosides. Both 12_H03-13 and 12_J03-18 cleaved CMU-xyloside, with the specificity constant of the 12_H03-13 wild-type enzyme being the same, within error, as that of the H03_E507A variant in which the GH8 activity was abated (Table 2), indicating that it was the GH43 domain that was responsible for the xylosidase activity. Surprisingly, none of the enzymes cleaved any of the other aryl glycosides tested (Supplementary Table 8), reinforcing the utility of these inherently more reactive chlorocoumarin glycosides for detection of previously unknown activities.

**Table 2:** Kinetic parameters  determined for purified GH43 enzymes with 6-chloro-4-methylumbelliferyl β-D-xyloside

Enzyme	K_M_(mM)	k_cat_(s^-1^)	k_cat/_K_M_ (s^-1^ mM^-1^)
12_J03-18	0.48 ± .06	0.22 ± .02	0.45 ± .09
12_H03-13 WT	0.19 ± .03	0.80 ± .06	4.2 ± 0 .7
12_H03-13_E507A	0.14 ± .01	0.73 ± .02	5.2 ± 0.4

**Fig. 3:**
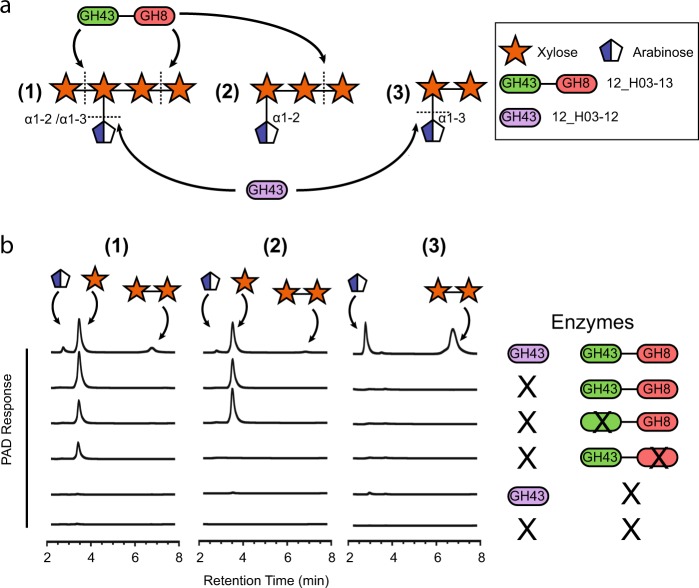
Activity of GH43s belonging to subfamilies 2 and 7 identified on fosmid 12_H03. **a** Schematic of the activities of the individual domains of 12_H03-13 and 12_H03-12 on arabinoxylans. These two enzymes were tested for activity on a mixture of 2^3^-α-L-arabinofuranosyl-xylotetraose and 3^3^-α-L-arabinofuranosyl-xylotetraose (**1**), 2^3^-α-L-arabinofuranosyl-xylotriose (**2**) and 3^2^-α-L-arabinofuranosyl-xylobiose (**3**). The GH8 domain of 12_H03-13 releases xylose from the reducing end of (**1**) and (**2**). The GH43 domain of 12_H03-13 releases xylose from the non-reducing end of (**1**). 12-H03-12, a GH43 belonging to subfamily 7 is able to release arabinose from only the oligomers containing an arabinose α-1-3-linkage. **b** High performance anion exchange chromatography with pulsed amperometric detection (HPAEC-PAD) analysis of the degradation of (**1**), (**2**) and (**3**) catalyzed by H03-12, H03-13, H03-13_E209A (GH43 domain mutant, denoted with an X on the GH43 domain) or H03-13_E507A (GH8 domain mutant, denoted with an X on the GH8 domain) and their combinations

The activities of enzymes 12_H03-12, 12_H03-13 and its variants were also tested on a set of arabinoxylan oligosaccharides. This revealed a synergistic degradation mechanism in which the GH43 domain of 12_H03-13 (subfamily 2) releases undecorated xylose from the non-reducing end of the oligosaccharides while the GH8 domain of 12_H03-13 (a reducing end xylose-releasing exo-oligoxylanase [Rex]) releases xylose from the reducing end of decorated oligosaccharides (Fig. 3). The activity displayed by 12_H03-13 is further complemented by GH43 12_H03-12 (subfamily 7), which cleaves α-1-3-linked arabinose decorations from arabinoxylans, releasing arabinose and xylobiose. This activity is only observed in the presence of 12_H03-13. This establishes the intriguing possibility that 12_H03-12 is activated by 12_H03-13. The xylobiose generated by these two enzymes appears to be resistant to further degradation by these enzymes. As GH8 Rex genes typically require at least a trisaccharide for activity, this domain is not expected to hydrolyze xylobiose. The GH43 domain of 12_H03-13 was expected to further degrade xylobiose, yet this is not the case, suggesting that the presence of an arabinose sidechain may be important for the xylosidase activity of this domain. This represents, to our knowledge, the first multi-domain protein containing both a GH43 and GH8 domain to be characterized and the first description of how these two domains function synergistically on arabinoxylan oligosaccharides converting them into arabinose and xylobiose. Collectively these results illuminate substrate specificity and activity of GH43 subfamilies 2, 7, and 28 within the context of the beaver fecal microbiome with direct relevance to lignocellulosic biomass conversion and host nutrition.

**Fig. 4:**
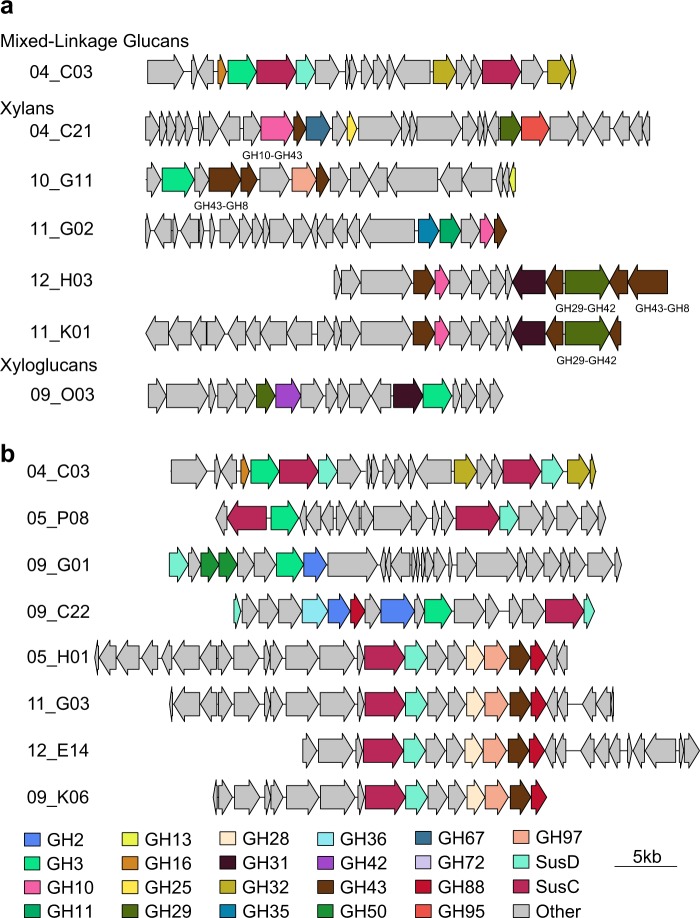
Gene organization of putative hemicellulose targeting fosmids and SusC/SusD-like encoding fosmids. **a** Fosmids with gene clusters that may target the hemicellulosic portion of plant biomass within the beaver diet. **b** SusC/SusD-like encoding fosmids. Putative glycoside hydrolases and SusC/SusD-like proteins are colored with the same scheme as Fig. 2. ORFs not annotated as a glycoside hydrolase, SusC-like, or SusD-like, are shown in gray. Fosmids Identical to 5_P08 have been omitted for simplicity

In addition to providing a route toward functional validation of predicted GH genes, active clone sequences contained information about the structural organization of GH gene cassettes. This has revealed several GH gene clusters that appear to target plant cell wall hemicelluloses (Fig. 4a). The fosmid 04_C03 contains a motif (GH16 and GH3 adjacent to SusC and SusD-like proteins) with synteny to a PUL recently shown to be active against mixed-linkage glucans [49]. Several fosmids (04_C21, 10_G11, 11_G02, 12_H03, and 11_K01) also appear to target xylans. The four fosmids 04_C21, 11_G02, 12_H03, and 11_K01 all harbor GH10 genes, which often act as endo-xylanases, and GH43s which may have exoxylanase activity. Furthermore, Fosmid 04_C21 contains a motif (GH10-GH43 (GH43 subfamily 12) protein followed by an additional GH43 (subfamily 1) and a GH67 with synteny to a gene cluster identified in *Bacteroides intestinalis* [50]. The GH10-GH43 homolog from *B. intestinalis* has endo-xylanase and arabinofuranosidase activity, which is able to release xylose, xylo-oligosaccharides and arabinose from arabinoxylans [50]. Although Fosmid 10_G11 lacks a GH10 it does contain a two domain GH43-GH8 gene, which we speculate may have similar activity to H03-13 in targetting arabinoxylan oligomers. The presence of a GH29 (a family of α-L-fucosidases), GH42 (a family cotaining β-galactosidases) and GH31 (a family containing α-xylosidases) on the three fosmids 09_O03, 12_H03, and 11_K01 leads us to speculate that these fosmids may target fucogalactoxyloglucan which is present in most dicots and gymnosperms [51, 52]

Moreover, we identified 15 fosmids spanning 5 identity groups containing Sus-like genes (SusC or SusD), leading indicators for the identification of PULs using an automated PUL prediction tool [53] (Fig. 4b). Eight of these clones exhibited near complete nucleotide identity (5_O06, 05_O07d, 5_O08, 5_P05, 5_P06, 5_P07, 5_P08, and 5_P12) and 4 clones shared near complete nucleotide identity specifically in the PUL interval (12_E14, 11_G03, 5_H01, and 9_K06). The remaining 4 clones (4_C03, 9_C22, and 9_G01) contained unique PUL intervals. Representative PULs from each identity group were compared to the RefSeq database to see if they are also found in sequenced microbial genomes. PULs from 9_C22 and 9_G01 exhibited 99% and 98% nucleotide identity respectively to distinct regions of the *Alistipes senegalensis* JC50 genome whereas the most common PUL represented by 5_P08 exhibited 99% nucleotide identity to the genome of *Alistipes finegoldii* DSM17242. The remaining identity groups exhibited less than 7% nucleotide identity to reference genomes, indicating previously unrecognized architectures. In addition to the fosmids which appear to target hemicelluloses mentioned above, 05_H01 and homologs appear to target pectic polymers as they contain a GH28 (a family containing polygalacturonases) and a GH88 which may target the unsaturated reducing ends generated by pectate lyases. The substrates targeted by the PULs present on fosmids 05_P08, 09_G01, and 09_C22 are not immediately apparent, and further biochemical characterization will be needed to reveal their activity.

Taken together, the PULs and gene clusters identified on fosmids appear to target many of the hemicellulosic components of plant cell walls, including glucuronylxylan, xyloglucan, and pectins, which would be present in hard woods. Some of the polymers which these gene cassettes likely act on, however, are not present in hardwoods, such as mixed-linkage glucans, which are mainly found in grasses, and arabinoxylans, which are present in grasses and softwoods. The ability to degrade these polymers may support host nutrition when preferential food sources are scarce. Future characterization of these PULs and gene clusters has potential to shed light on combinatorial biomass deconstruction within the beaver microbiome at the population and community levels of organization [54–57].

Beavers have been described as nature’s engineers due to their prolific capacity to reshape forest ecosystems into ponds and meadows, raising groundwater levels and creating new habitats for diverse plants and animals. Beyond their capacity to transform landscapes, beavers are extremely efficient consumers of woody biomass relying on bark, shoots, leaves, and other fibers from hardwood deciduous trees as primary nutritional resources. The current study opens a functional metagenomic window on the capacity of the beaver fecal microbiome to deconstruct woody plants into soluble sugars supporting host nutrition. Although beavers are classified as xylotrophic organisms, their microbiome composition is most similar to those of hindgut-fermenting mammals dominated by Bacteroidetes and Firmicutes. However, the specific strains inhabiting the beaver fecal microbiome harbor abundant and diverse genomic adaptations underlying woody biomass deconstruction, particularly xylan substrates that can be funneled into central metabolism. These adaptations are distributed among and between different populations within the fecal microbiome and encompass multiple GH43 subfamilies (including one containing both GH8 and GH43 domains) that synergize in combination. Several of these subfamilies are encoded in PULs representing previously unknown genomic architectures. Targeted conversion screens identified potential substrates for three GH43 subfamilies expanding our mechanistic understanding of this important group of CAZymes. Interestingly we did not identify an abundance of fosmid genes encoding cellulases, indicating the potential for overexpression by specialized strains or compartmentalization of function along the beaver gastrointestinal tract.

The panoply of genes encoding enzymes with hemicellulose degrading activities is presumably required to tackle the complex structures of hardwood biomass in which glucan and xylan backbones are extensively decorated with appended sugars. The presence of two-domain enzymes endows the beaver fecal microbiome with synergistic capacity to efficiently degrade the specific linkages present within the hemicellulose. Not only does this liberate monosaccharides, but the degradation of hemicellulose scaffolds also exposes the underlying cellulose fibers for digestion by the cellulase repertoire, enhancing conversion efficiency. Taken together these results provide a unique perspective on the modular domain architecture and functional specialization driving combinatorial biomass deconstruction in the beaver fecal microbiome. It will be of interest to determine the extent to which beavers combine compartmentalization and sequential biomass deconstruction with combinatorial enzymatic activities acting on lignocellulosic substrates. These deconstruction principles can in turn be used in the design of enhanced enzyme mixtures and whole cell biocatalysts tuned to xylan inputs in modern biorefining ecosystems.

## Methods

### Sample collection

Fecal samples were collected in 50 mL Falcon tubes on April 12th, 2012 from two beavers that were being cared for at the Critter Care Wildlife Society located in Langley, British Columbia, Canada. Animals were fed branches from a variety of woody plant species, native to the Pacific Northwest. Due to the difficulty of obtaining fresh fecal matter, as beavers defecate underwater, samples were collected from material that had accumulated at the enclosure water outflow grating, within 12 h of cleaning. The enclosure was open to the environment and not heated. The temperature fluctuated between 7 and 15 °C in the time before sample collection. As both beavers shared the same enclosure, it was not possible to identify which animal the samples came from. Samples were frozen in a slurry of dry-ice and ethanol and transported to the laboratory on dry-ice and were stored at –80 °C.

### DNA extraction

High molecular weight DNA was extracted from four grams of beaver feces as described previously [58]. Extracted DNA was purified by isopropanol precipitation and quantified using the PicoGreen assay (Invitrogen).

### PCR amplification of ribosomal SSU rRNA gene sequences

Following DNA isolation, the V6-V8 region of the small subunit ribosomal RNA (SSU rRNA) gene was PCR amplified with the universal three-domain primers 926F and 1392R containing barcodes for pooled sequencing. Duplicate reactions were pooled and purified and then quantified. Samples were diluted to 10 ng/µL and pooled in equal concentrations. See supplemental methods for further details.

### SSU rRNA amplicon sequencing and analysis

SSU rRNA amplicon pools were sent to the Génome Québec Innovation Centre at McGill University for 454 pyrosequencing using the Roche 454 GS FLX Titanium platform. QIIME [59] was used to quality control and analyze the amplicon sequences, which were then queried against the SILVA database version 111 [20]. See supplementary information for detailed methods.

### Metagenomic sequencing and analysis

Genomic DNA was sent to the Génome Québec Innovation Centre at McGill University for whole genome shotgun sequencing using the Roche 454 GS FLX Titanium platform. 454 sequence data were assembled using MIRA version 4.0.2 [27]. Open reading frames (ORFs) were predicted using Prodigal [60] implemented in the MetaPathways pipeline [28]. The assembled metagenome yielded 151,180 ORFs >180 nucleotides in length annotated using LAST [61] implemented in the MetaPathways pipeline based on queries of KEGG [62], COG [63], RefSeq [64], MetaCyc [65], and CAZy [30] databases. Metagenomic data from previous studies [2, 31, 32, 66] were downloaded from the RAST online database and used for comparison.

Metagenomic binning of the MIRA contigs and the 53 assembled fosmid sequences was performed using MaxBin 2.0 [34]. The default MaxBin abundance estimation workflow was unable to estimate abundance of these contigs due to the single-end nature of pyrosequenced reads, therefore abundance files for binning were generated by BWA MEM and a custom script for generating RPKM values in a compatible format [67]. Apart from this, default parameters were used resulting in 30 metagenome-assembled genomes (MAGs). All 51 fosmids were binned across 12 different MAGs. MAGs were refined using the Anvi’o metagenomics workflow [35]. Centrifuge was used to assign taxonomy to contigs to assist with manual MAG refinement within this workflow [68]. Additionally, the ‘--cluster-contigs’ flag was used to estimate the relatedness of these contigs without abundance information from multiple samples and a minimum contig length of 1,500 bp was enforced to reduce marker gene redundancy from short contigs. checkM was used to evaluate the final completeness and contamination of these MAGs [69], yielding 8 medium-quality (completeness >50% and contamination <10%) and 22 low-quality MAGs [36]. Taxonomy was assigned to the medium-quality MAGs using Phylosift by taking the deepest taxonomic assignment with >70% of the taxonomic annotations [37], see Table S4.

### Fosmid library construction and screening

Genomic DNA was further purified by cesium chloride gradient ultracentrifugation prior to library creation [70]. A large insert library was constructed as described previously [71] using the CopyControl™ Fosmid Library Production Kit with pCC1FOS™ Vector Kit (EpiCentre). This resulted in a library containing 4608 clones. Functional screening was performed according to procedures by Mewis et al. [42] with modifications. Screening was carried out in phosphate buffer (25 mM sodium phosphate, pH 6.0), containing 100 µM each of the three fluorogenic substrates (6-chloro-4-methylumbelliferyl β-cellobioside, 6-chloro-4-methylumbelliferyl β-xylobioside, and 6-chloro-4-methylumbelliferyl β-D-xylopyranoside). Screening was performed at a temperature of 37 °C, which is the body temperature of *Castor canadensis* [72]. Fosmids chosen for sequencing (z-score > 3 for each substrate) were rearrayed using an automated colony-picking robot (Qpix2, Molecular Devices), into a 96 well plate (Costar 3370) containing 200 µL of LB chloramphenicol (12.5 µg/mL) and 10% glycerol. This master plate was incubated overnight at 37 °C and then stored at −80 °C.

The frozen master plate was then used to assay the clones against a panel of eleven different fluorogenic substrates, see supplemental methods for detailed description.

### Fosmid sequencing and analysis

The 96 well master plate was used to inoculate a 96 deep-well plate (Costar) containing 1.65 mL LB with chloramphenicol (12.5 µg/mL) and arabinose (100 µg/mL). This plate was incubated with shaking (37 °C, 320 rpm) for 20 h, then centrifuged at 1,500 ×*g* for 10 min and the supernatant was decanted. Fosmids were purified from the pelleted cells using a Montage Plasmid MiniprepHTS 96 Kit (Millipore), treated with PlasmidSafe ATP-dependent DNAse (Epicentre) and quantified using the PicoGreen assay (Invitrogen). Purified DNA was prepared for sequencing on the Illumina MiSeq platform using Nextera XT library preparation kit and 96 sample Nextera V1 index kit. Samples were sequenced using paired end 150 bp reads (2 × 150 bp mode). Fosmid sequence quality control and assembly are detailed in the supporting methods. Assembled fosmid contigs were were annotated in the same manner as the assembled metagenome. The resulting RefSeq output files were imported into MEGAN [73] as a way of assigning taxonomy to each fosmid according to the LCAstar algorithm [74].

### Cloning and expression of GH43 genes

One GH43 gene from each of the uncharacterized subfamilies 2, 7, and 28 was chosen for cloning, expression and characterization. The three genes (12_H03-12, 12_H03-13, 12_J03-18), were inserted into a pET28 vector by use of the polymerase incomplete primer extension method [75]. The mutant enzymes H03-13_E507A and H03-13_E209A were produced by means of modified QuikChange mutagenesis [76]. PCR parameters and primers used are detailed in the supplemental methods. The His-tagged proteins were purified by nickel-metal affinity chromatography, as described in the supplemental methods.

### GH43 activity assays

Purified GH43 enzymes were tested against a variety of glycosidase substrates, see supplemental information for detailed methods.

### Data accession

Data is deposited in the NCBI BioProject portal (Bioproject ID: PRJNA261082), for assembled metagenomic reads (BioSample ID: SAMN04122864), unassembled metagenomic reads (BioSample ID: SAMN03389401), functionally identified fosmids (Biosample ID: SAMN03389402), and pyrotags (Biosample ID: SAMN03389403).

## Electronic supplementary material

Supplementary Methods

Figure S1

Figure S2

Figure S3

Figure S4

Figure S5

Figure S6

Supplemental Figure Captions

Table S1

Table S2

Table S3

Table S4

Table S5

Table S6

Table S7

Table S8
