# Supplementary material for: Metagenomics reveals functional synergy and novel polysaccharide utilization loci in the Castor canadensis fecal microbiome
Source: ISME J. 2018 Jul 16;12(11):2757–69. doi: 10.1038/s41396-018-0215-9 (PMC6193987; doi:10.1038/s41396-018-0215-9)
Supplement: Supplementary file 1 — Supplementary Methods [file 41396_2018_215_MOESM1_ESM.pdf]

Metagenomics Reveals Functional Synergy and Novel Polysaccharide Utilization Loci in the *Castor canadensis* Fecal Microbiome

Zachary Armstrong, Keith Mewis, Feng Liu, Connor Morgan-Lang, Melanie Scofield, Evan Durno, Hong Ming Chen, Kevin Mehr, Stephen G. Withers and Steven J. Hallam

Supplementary Methods

PCR amplification of ribosomal SSU rRNA gene sequences

Following DNA isolation, the V6-V8 region of the small subunit ribosomal RNA (SSU rRNA) gene was PCR amplified with the universal three-domain primers 926F (5′-AAA CTY AAA KGA ATT GAC GG-3′) and 1392R (5′-ACG GGC GGT GTG TRC-3′). Reverse primer sequences were modified to include the 454 adaptor sequence and a 5 base-pair (bp) barcode for multiplexing during sequencing. Reactions were run in duplicate under the following PCR conditions: initial denaturation cycle at 95 °C for 3 minutes; 25 cycles of 95 °C for 30 seconds, primer annealing at 55 °C for 45 seconds, and extension at 72 °C for 90 seconds; and a final extension cycle at 72 °C for 10 minutes. Each 50 µL reaction contained: 1-10 ng template DNA, 0.6 µL Taq polymerase (Bioshop Canada Inc., 5 U/µL), 5 µL 10X reaction buffer, 4 mM MgCl2, 0.4 mM of each dNTP (Invitrogen), 200 nM each of forward and bar-coded reverse primers, and 33.4 µL nuclease free water (Fisher). Duplicate reactions were pooled and purified using a QIAquick PCR Purification Kit® (Qiagen) and quantified using the PicoGreen assay (Invitrogen). Samples were diluted to 10 ng/µL and pooled in equal concentrations.

SSU rRNA amplicon sequencing and analysis

The software package Quantitative Insights Into Microbial Ecology (QIIME) (Caporaso et al., 2010) was used to analyze a total of 14,050 SSU rRNA amplicon sequences. As a quality

control step, sequences with quality scores less than Q25, those containing ambiguous bases, or identified homopolymer runs, or chimeric sequences, or with length less than 200 bp were removed. The remaining 12,579 high quality sequences (average length = 454 bp) were clustered at the 97 % identity threshold with a maximum e-value cut-off of 1 x 10^{-10} using UCLUST, implemented in QIIME software (Edgar, 2010). Singletons were omitted from downstream analyses, leaving 404 operational taxonomic units (OTUs) made up of 12,128 pyrotag sequences. Taxonomic assignment for each OTU cluster was performed using the Basic Local Alignment Tool (BLAST) (Altschul et al., 1997) and the SILVA database version 111 (www.arb-silva.de) (Quast et al., 2013) with a confidence level of 0.8 and a maximum e-value cut-off of 1 x 10^{-3}. OTU abundance was normalized to the total number of reads recovered, and expressed as a normalized percentage for analysis.

Fosmid library screening

The frozen master plate was used to inoculate a deep well plate (Costar) containing 0.8 mL of LB containing chloramphenicol (12.5 μg/mL) and arabinose (100 μg/mL), which was then incubated at 37 °C for 18 hours with shaking at 225 rpm. Cells were harvested by centrifugation (3220 x g, 20 min). Supernatant was decanted, cell pellets were re-suspended in 200 μL of buffer (50 mM sodium phosphate, 10 mM NaCl, pH 6.0) and OD600 was recorded. This cell suspension was added the same volume of lysis buffer (50 mM sodium phosphate, 10 mM NaCl, 2 % triton, 0.5 mg/mL lysozyme, cOmplete Protease Inhibitor- EDTA free (Roche), pH 6.0) and incubated for 1h at room temperature. Activity assays were performed in 96-well plates (Costar) containing 40 mM sodium phosphate, 200 μM substrate and 20 μL of lysate. Substrates assayed for activity were: 6-chloro-4-methylumbelliferyl β-cellobioside, 6-chloro-4-methylumbelliferyl β-xylobioside, and 6-chloro-4-methylumbelliferyl β-D-xylopyranoside,

methylumbelliferyl β-cellobioside, methylumbelliferyl β-D-glucopyranoside, methylumbelliferyl β-xylobioside, methylumbelliferyl β-D-xylopyranoside, methylumbelliferyl β-lactopyranoside, methylumbelliferyl β-D-galactopyranoside, methylumbelliferyl β-D-mannopyranoside, methylumbelliferyl α-L-arabinofuranoside and methylumbelliferyl N-acetyl-β-D-glucosaminide. Reactions were set up on a Beckman Coulter Biomek® FX workstation and run in triplicate at room temperature. Samples (10 μL) were taken at 1, 2, 4, 6 h, quenched with stop buffer (1 M glycine, pH=10.4) and analyzed by fluorescence spectroscopy on a Beckman Coulter DTX-880 Multimode Detector (λex = 365, bandwidth 25nm, λem = 465, bandwidth 35 nm).

Fosmid sequencing and analysis

FastQ sequences were obtained from the sequencer and quality was assessed using FastQC (Andrews, 2010). Raw sequences were trimmed to Q30 quality, and residual contaminating *E. coli* genomic DNA was removed by alignment to the *E. coli* K12 reference genome using the bwa aligner (Li and Durbin, 2009). Trimmed reads were assembled at a range of kmer values (64 to 160) using ABySS (Simpson et al., 2009) and the kmer value that produced the fewest contigs of appropriate size (25 - 40 kb) was selected. The presence of pCC1 vector sequence at ends of fosmids signaled the proper contig to select. Wells that did not produce contigs with pCC1 vector present were end sequenced and compared to all contigs produced from that well to identify the correct sequence.

Cloning and expression of GH43 genes

Sub-cloning: Each PCR reaction contained 10 μL of Phusion reaction buffer, 1.5 μL of dNTPs (10 mM), 1 μL forward primer (10 μM), 1 μL reverse primer (10 μM) 2 μL of template DNA (5

ng/μL) 0.5 μL Phusion polymerase and 34 μL of water. The insert PCR was performed with the following parameters: Initial denaturation at 95 °C for 2 minutes followed by 25 cycles of denaturation at 95 °C (30 s), annealing between 57 °C and 70 °C (30 s) and extension at 72 °C (1 min). Insert primers used were:

H03-12.I.PIPE.F (ctttaagaaggagatataccatgCAGGTGGGGCAACCCTGGAT),
H03-12.I.PIPE.R (gatctcaatggtgatggtgatggtgAGGTTCCCTCCTCATCCTCC)
H03-13.I.PIPE.F (ctttaagaaggagatataccatgCAAAACCCGCTCATCCACTC),
H03-13.I.PIPE.R (gatctcaatggtgatggtgatggtgTTTTACATCCACAGTGATATTCC),
12_J03.I.PIPE.F (ctttaagaaggagatataccATGAAAACCTACTGCAACCCG), and
12_J03.I.PIPE.R (gatctcaatggtgatggtgatggtgGCCCTCCATCTTTACAATTTC).

Vector PCR was performed as above, except the annealing temperature was 55 °C and the extension time was 3.5 minutes. Vector Primers used were:

H03-12.V.PIPE.F (AGGATGAGGAGGGAACCTcaccatcaccatcaccat)
H03-12.V.PIPE.R(AATCCAGGGTTGCCCCACCTGcatggtatatctccttcttaaag)
H03-13.V.PIPE.F(ATCACTGTGGATGTAAAAcaccatcaccatcaccat)
H03-13.V.PIPE.R(CGAGTGGATGAGCGGGTTTTGcatggtatatctccttcttaaag)
12_J03.V.PIPE.F(ATTGTAAAGATGGAGGGCcaccatcaccatcaccat)
12_J03.V.PIPE.R(GCGGGTTGCAGTAGGTTTTcatggtatatctccttcttaaag)

PCR products were mixed and transformed into DH5α cells, plasmids were sequence verified, then transformed into BL23 (DE3) cells for expression.

Mutagenesis: PCR was first performed for 12 cycles with one of the sense or anti-sense primers these two reactions were subsequently pooled and an additional 16 cycles of PCR were performed. Primers used were:

H03-13_E507A.F (GATGTGCGCACCGCCGGAATGTCATAC)
H03-13_E507A.R(GTATGACATTCCGGCGGTGCGCACATC)
H03-13_E209A.F(CGAAGGCTTCAAGGCAGGGCCCTTCGCCTTC) and
H03-13_E209A.R(GAAGGCGAAGGGCCCTGCCTTGAAGCCTTCG).

PCR products were digested with DpnI and transformed into chemically competent DH5α cells. Upon full gene sequence verification plasmids were transformed into a BL21 (DE3) expression host.

Purification: Proteins were purified with use of polyhistidine tags and Ni-NTA resin columns. Cultures of 50 mL LBE-5052 (Studier, 2005), containing 50 µg/L of kanamycin were inoculated with the expression host and cells were grown for 18 hours at 37 °C (12_H03-12 and 12_J03-18) or 30 ºC (12_H03-13, H03-13_E507A and H03-13_E209A) with shaking. Cultures were centrifuged (3220 x g, 4 °C, 20 min), the supernatant was removed and cell pellets were stored at -80 °C until purification. To purify proteins 2.5 mL of lysis mix (1 x BugBuster (Novagen), 20 mM HEPES, 300 mM NaCl, 20 mM Imidazole, pH 7.0) was used to re-suspend thawed cell pellets. The resulting suspension was incubated at 20 °C for 20 minutes, after which the lysate was clarified by centrifugation (3220 x *g*, 4 °C, 20 min) and loaded onto columns containing 1 mL of HisPur resin (Thermoscientific). Columns were washed with 20 mL of Buffer A (20 mM HEPES, 300 mM NaCl, 20 mM Imidazole, pH 7.0) and protein was eluted with 4 mL of Buffer B (20 mM HEPES, 300 mM NaCl, 500 mM Imidazole, pH 7.0). Proteins were buffer exchanged into storage buffer (20 mM HEPES, 300 mM NaCl, pH 7.0) with Amicon 30 kDa filter columns and stored at 4 °C. Protein concentrations were determined based on absorbance at 280 nm.

GH43 Activity Assays

Each purified enzyme was tested for activity on the following model substrates: p-nitrophenyl β-D-xylopyranoside, 4-methylumbelliferyl β-D-xylopyranoside, 6-chloro-4-methylumbelliferyl β-D-xylopyranoside, p-nitrophenyl β-D-xylopyranoside, p-nitrophenyl α-L-arabinofuranoside, 4-methylumbelliferyl α-L-arabinofuranoside. Purified enzyme was added (final concentrations of 200 nM) to a solution of 100 µM substrate, 50 mM HEPES, 50 mM NaCl, pH 7.0. These assays were incubated at 37 °C for 18 hours after which absorbance (λ = 400 nm) and fluorescence (λ_{ex} = 365 nm, λ_{em} = 450nm) were detected with a BioTek synergy H1 plate reader. Kinetic parameters were determined using 6-chloro-4-methylumbelliferyl β-D-

xylopyranoside. Assays were performed in 96 well plates (Corning 3370) containing the substrate (2.5 μM – 100 μM), buffer (50 mM HEPES, 50 mM NaCl, pH 7.0) and purified enzyme. Reactions were performed at 30 °C and fluorescence (λ_{ex}= 365 nm and λ_{em}= 450 nm, gain = 65) was monitored using a Synergy H1 plate reader (BioTek). The quantity of fluorophore generated was determined by means of a calibration curve of 6-chlorocoumarin within an identical buffer system. All reactions were performed in triplicate. Rate measurements were used to calculate kinetic parameters with the software program GraFit 7.0 software.

Enzyme activity was also determined on the oligosaccharides, 3^2-α-L-arabinofuranosyl-xylobiose (A3X), 2^3-α-L-arabinofuranosyl-xylotriose (A2XX) and a mixture of 2^3-α-L-arabinofuranosyl-xylotetraose and 3^3-α-L-arabinofuranosyl-xylotetraose (XA^3XX/XA^2XX). Purified enzymes (final concentration of 0.5 μM per enzyme) were added to a solution of 4 mM substrate in HEPES buffer (50 mM HEPES, 50 mM NaCl, pH 7.0). Assays were incubated at 25 °C for 18 hours, then subsequently boiled for 10 min to inactivate the enzymes. Products were analyzed with the use of a high performance anion-exchange chromatography equipped with a pulsed amperometric detector. This system was equipped with a CARBOPAC™ PA-200 analytical anion exchange column (Dionex). The elution conditions were: 0-4 min 20 mM NaOH; 4-13 min, 20 mM NaOH with a 0 – 84 mM sodium acetate gradient; 13-14 min, 20 mM NaOH with a 84 – 120 mM sodium acetate gradient; 14-16 min with 20 mM NaOH and 120 mM sodium acetate. The standards used to identify the chromatographic peaks were arabinose, xylose, xylobiose, and xylotetraose.

Supplementary References

Altschul, S. F., Madden, T. L., Schäffer, A. A., Zhang, J., Zhang, Z., Miller, W., et al. (1997). Gapped BLAST and PSI-BLAST: a new generation of protein database search programs. Nucleic Acids Research, 25, 3389-3402.

Andrews, S. (2010). FastQC: A quality control tool for high throughput sequence data. http://www.bioinformatics.babraham.ac.uk/projects/fastqc/.

Caporaso, J. G., Kuczynski, J., Stombaugh, J., Bittinger, K., Bushman, F. D., Costello, E. K., et al. (2010). QIIME allows analysis of high-throughput community sequencing data. Nat Meth, 7, 335-336.

Edgar, R. C. (2010). Search and clustering orders of magnitude faster than BLAST. Bioinformatics, 26, 2460-2461.

Li, H. & Durbin, R. (2009). Fast and accurate short read alignment with Burrows,ÄìWheeler transform. Bioinformatics, 25, 1754-1760.

Quast, C., Pruesse, E., Yilmaz, P., Gerken, J., Schweer, T., Yarza, P., et al. (2013). The SILVA ribosomal RNA gene database project: improved data processing and web-based tools. Nucleic Acids Research, 41, D590-D596.

Simpson, J. T., Wong, K., Jackman, S. D., Schein, J. E., Jones, S. J. M. & Birol, Ƒ. N. ß. (2009). ABySS: A parallel assembler for short read sequence data. Genome Research, 19, 1117-1123.

Studier, F. W. (2005). Protein production by auto-induction in high density shaking cultures. Protein Expr Purif, 41, 207-34.
