# Supplementary figures and images for: Metagenomics reveals functional synergy and novel polysaccharide utilization loci in the Castor canadensis fecal microbiome
Source: ISME J. 2018 Jul 16;12(11):2757–69. doi: 10.1038/s41396-018-0215-9 (PMC6193987; doi:10.1038/s41396-018-0215-9)

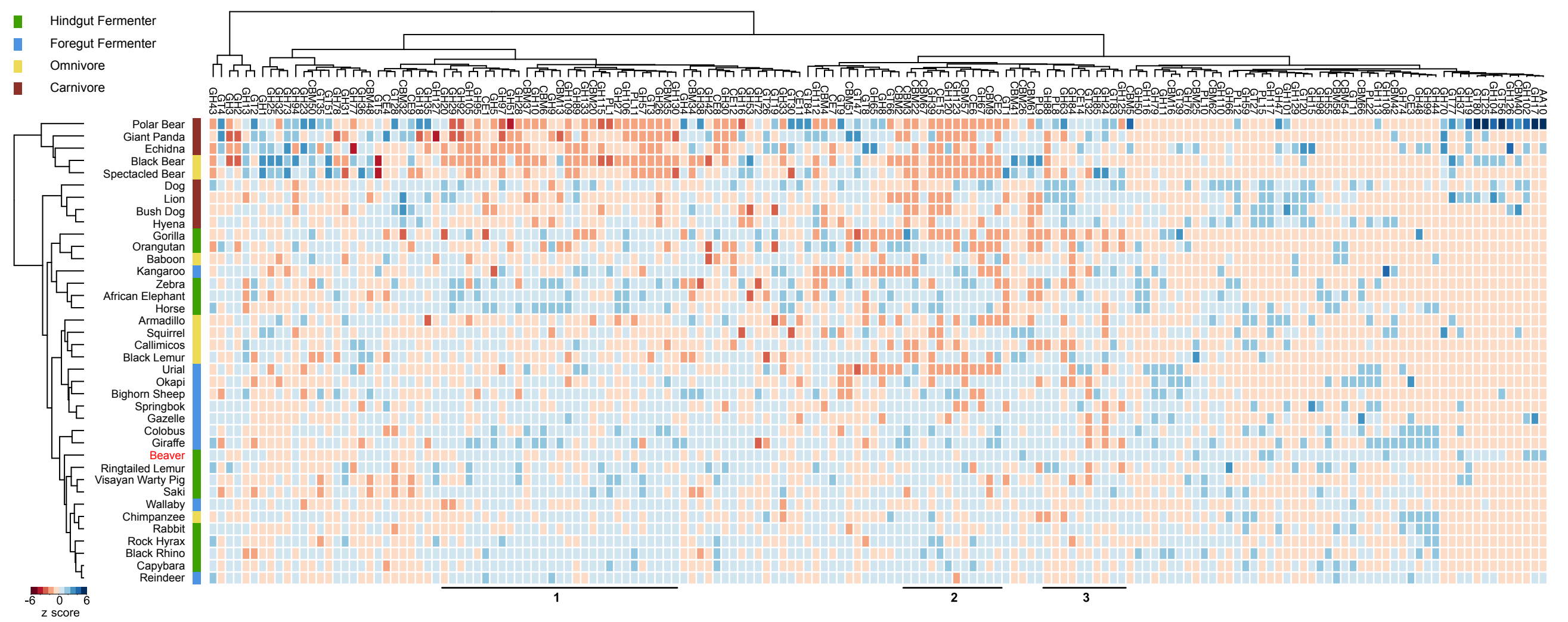

Supplement: Supplementary file 2 — Figure S1 [file 41396_2018_215_MOESM2_ESM.pdf]

450nm Fluorescence

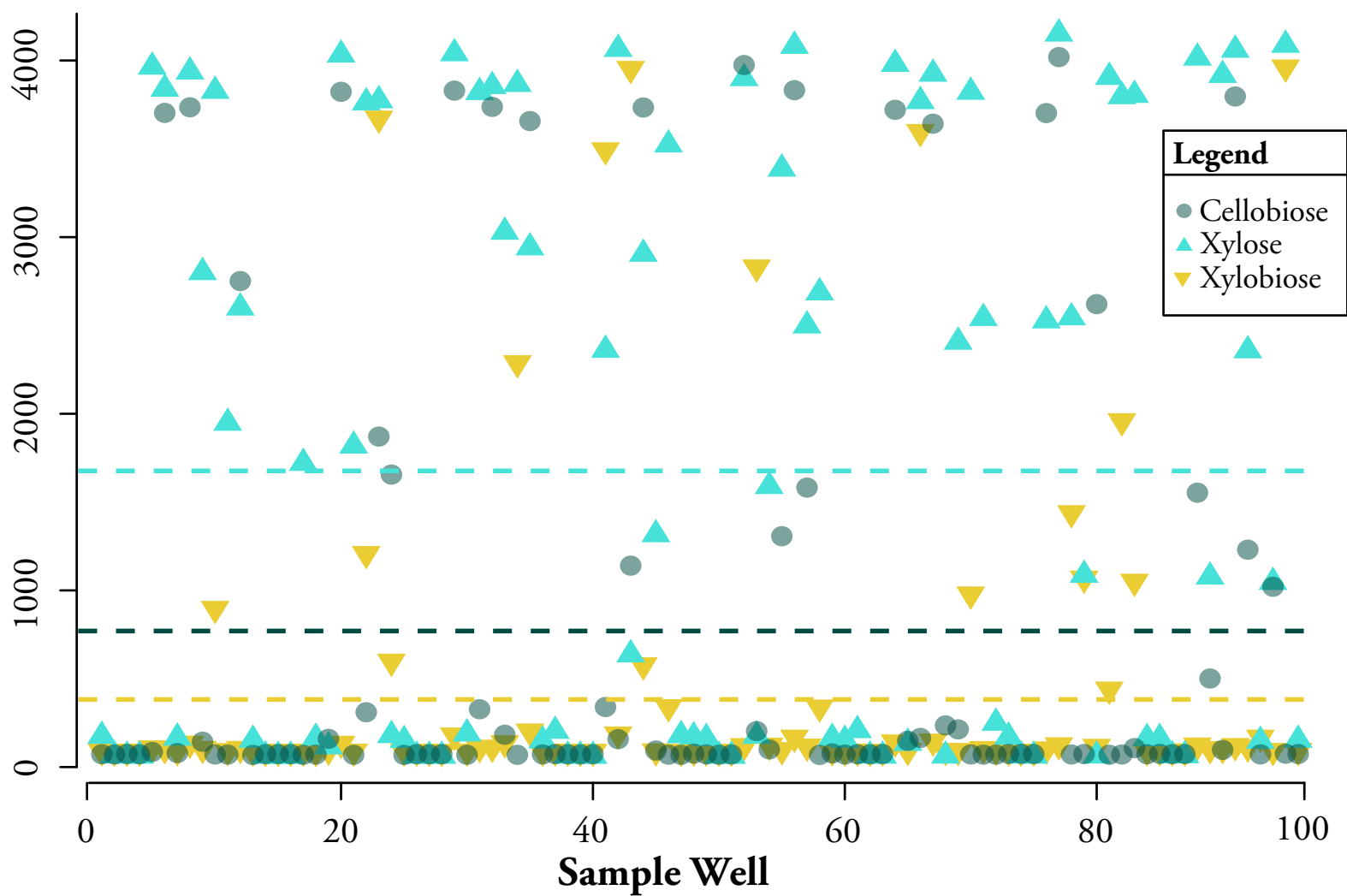

Supplement: Supplementary file 4 — Figure S3 [file 41396_2018_215_MOESM4_ESM.pdf]

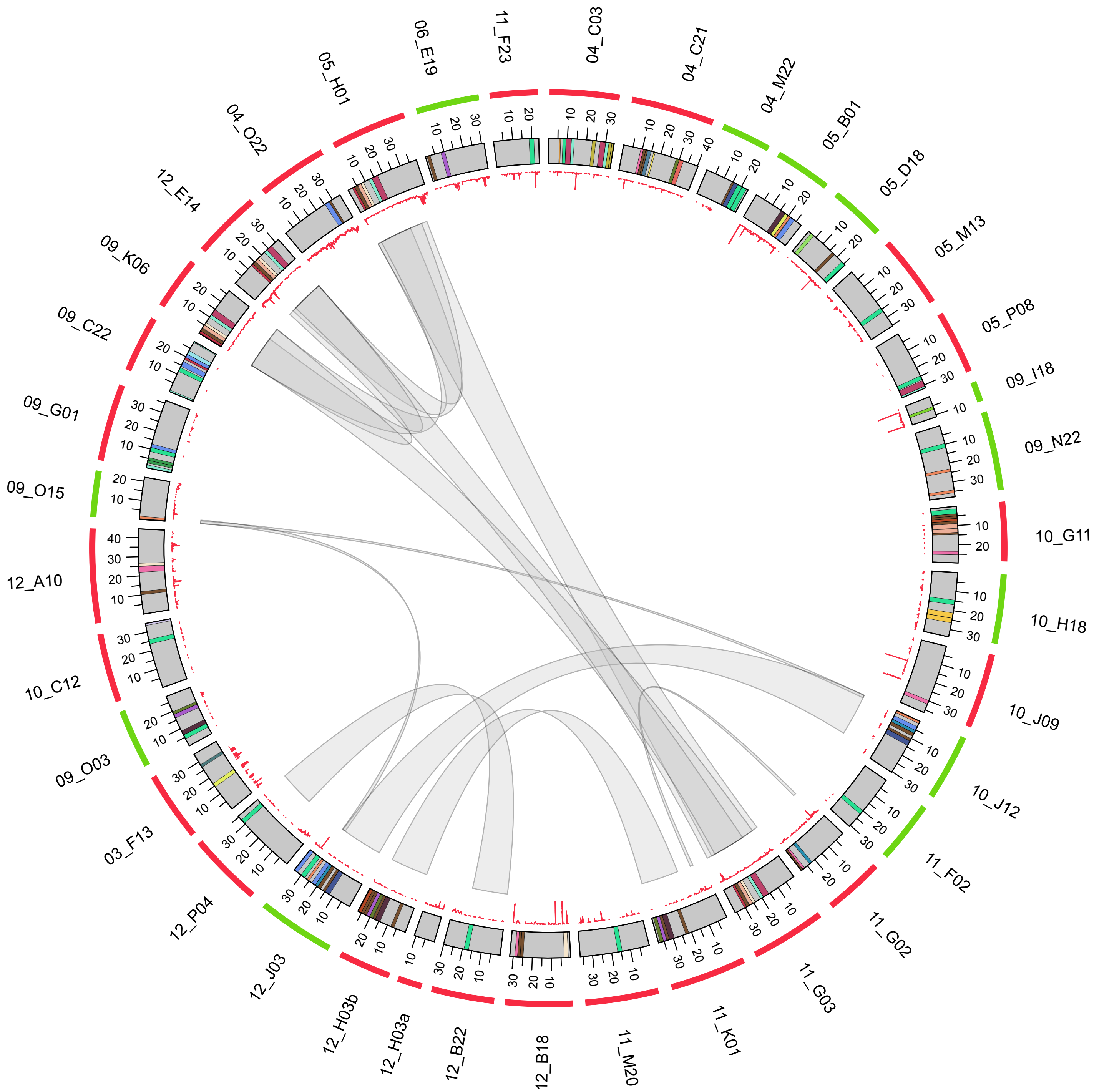

Supplement: Supplementary file 6 — Figure S5 [file 41396_2018_215_MOESM6_ESM.pdf]

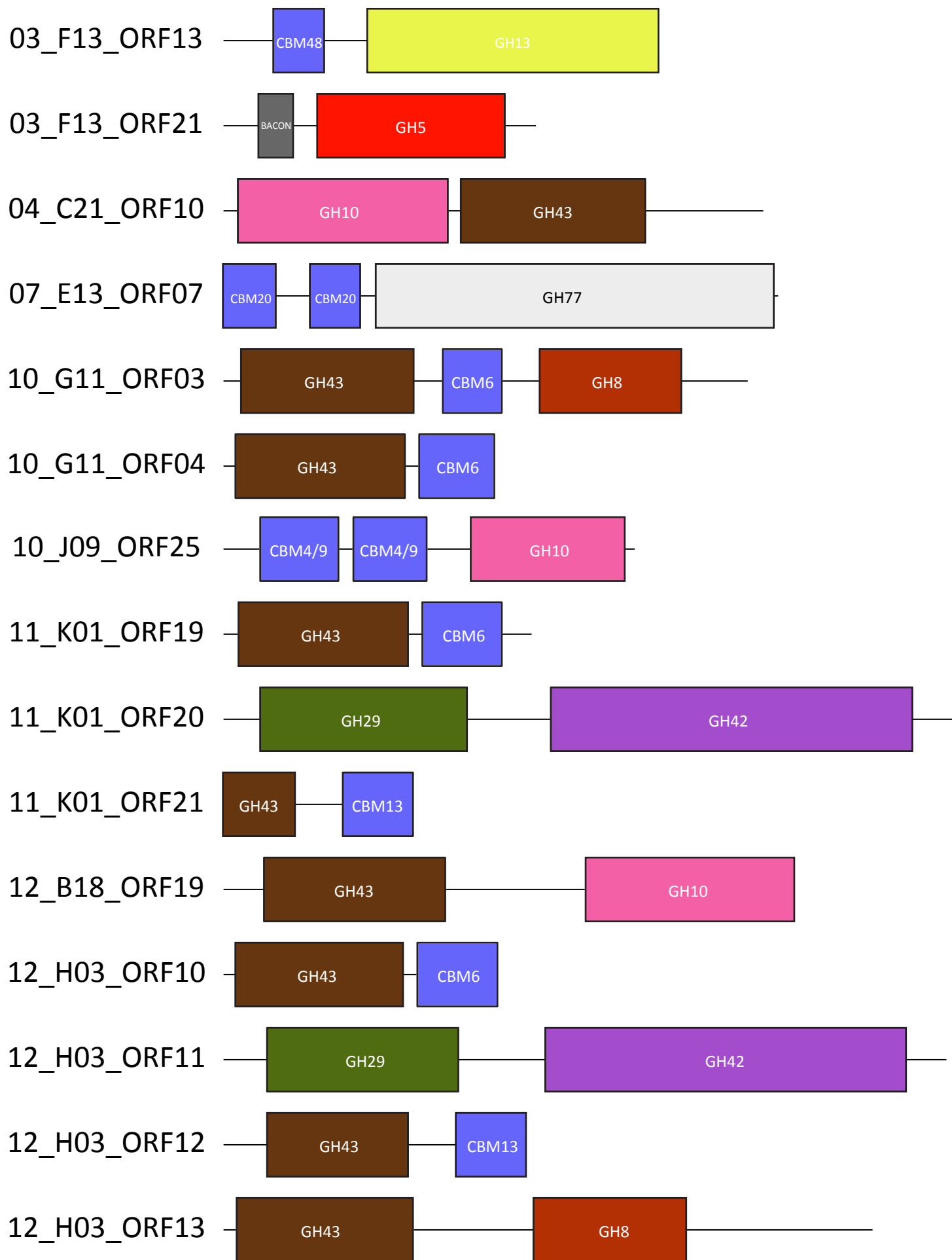

Supplement: Supplementary file 7 — Figure S6 [file 41396_2018_215_MOESM7_ESM.pdf]
