# Supplementary material for: Metagenomics reveals functional synergy and novel polysaccharide utilization loci in the Castor canadensis fecal microbiome
Source: ISME J. 2018 Jul 16;12(11):2757–69. doi: 10.1038/s41396-018-0215-9 (PMC6193987; doi:10.1038/s41396-018-0215-9)
Supplement: Supplementary file 3 — Figure S2 [file 41396_2018_215_MOESM3_ESM.pdf]

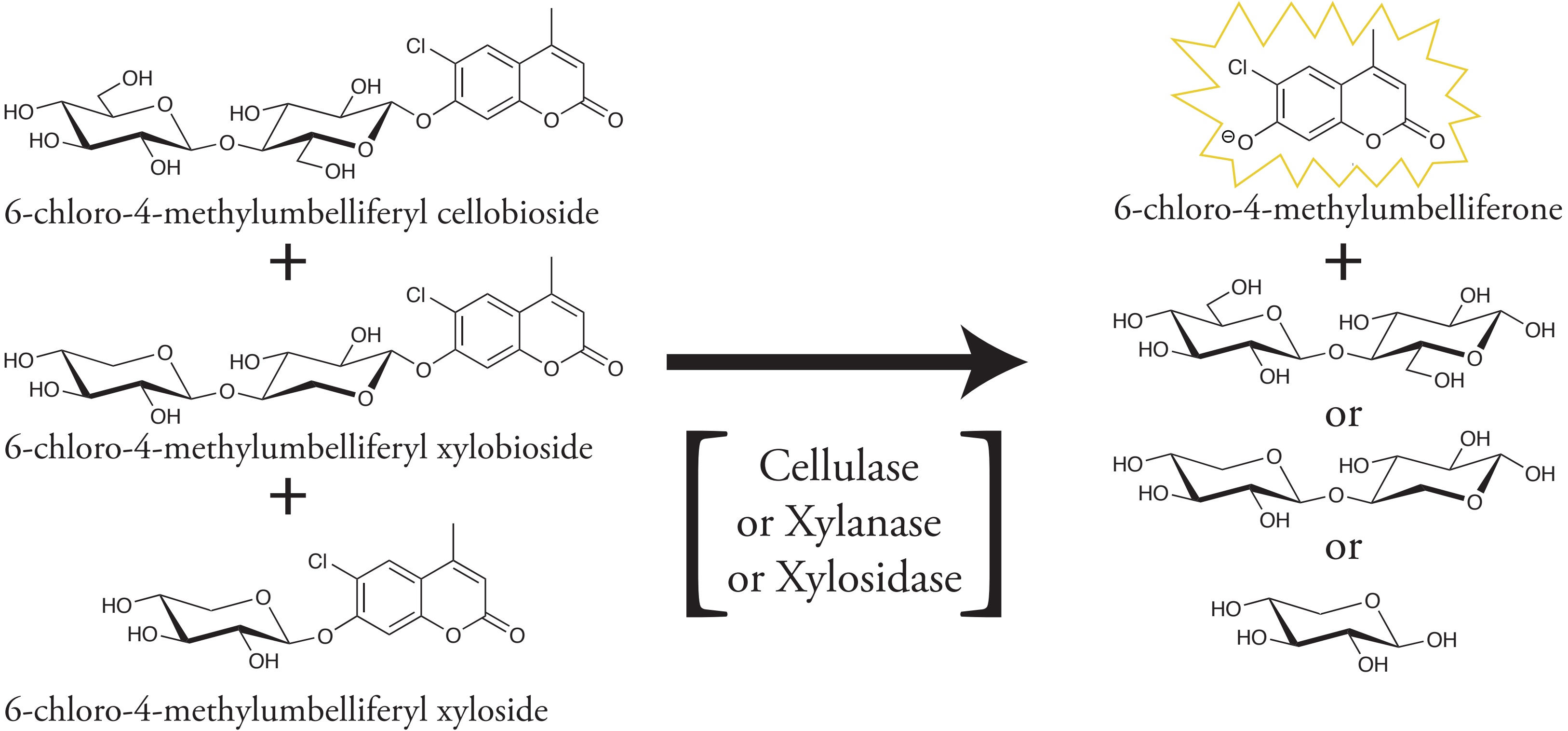
6-chloro-4-methylumbelliferyl cellobioside
+
6-chloro-4-methylumbelliferyl xylobioside
+
6-chloro-4-methylumbelliferyl xyloside
Cellulase
or Xylanase
or Xylosidase
6-chloro-4-methylumbelliferone
+
or
or
Cl
HO
OH
O
