# Supplementary material for: Metagenomics reveals functional synergy and novel polysaccharide utilization loci in the Castor canadensis fecal microbiome
Source: ISME J. 2018 Jul 16;12(11):2757–69. doi: 10.1038/s41396-018-0215-9 (PMC6193987; doi:10.1038/s41396-018-0215-9)
Supplement: Supplementary file 5 — Figure S4 [file 41396_2018_215_MOESM5_ESM.pdf]

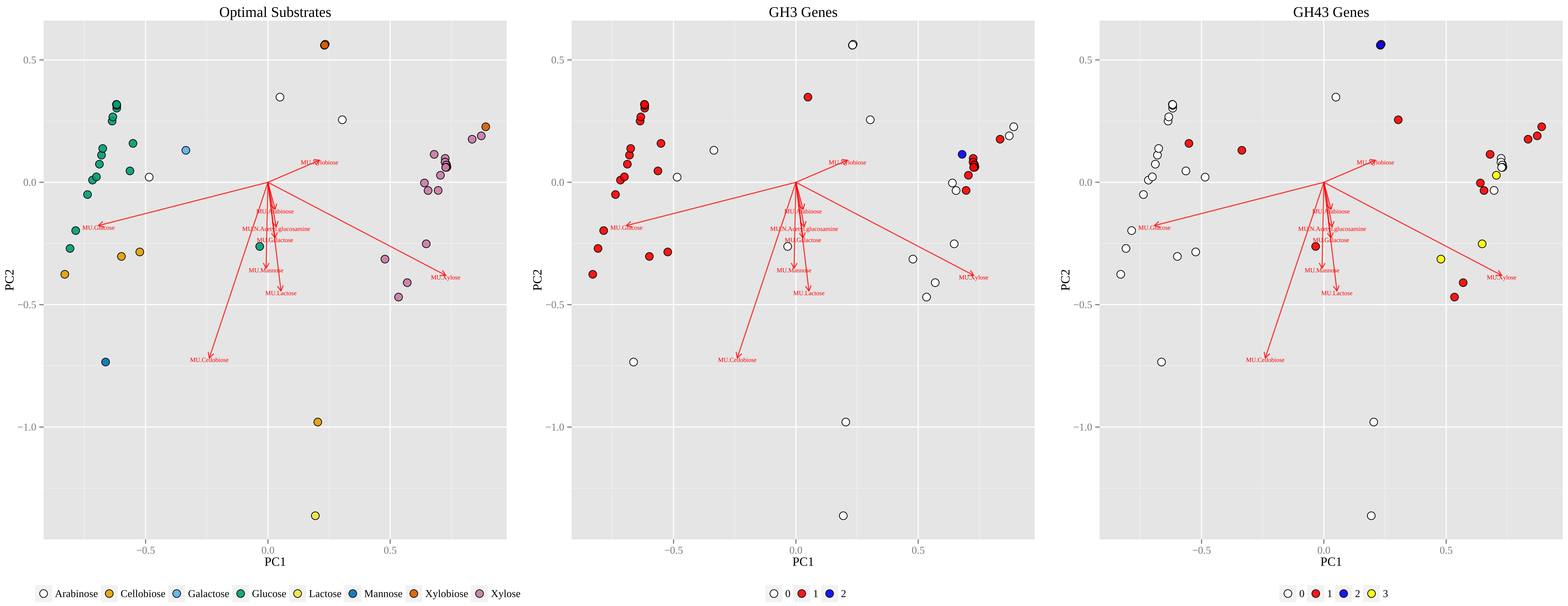

Optimal Substrates
GH3 Genes
GH43 Genes
PC1
PC2
0.5
0.0
−0.5
−1.0
MU.Xylobiose
MU.Arabinose
MU.N.Acetyl.glucosamine
MU.Galactose
MU.Glucose
MU.Mannose
MU.Lactose
MU.Xylose
MU.Cellobiose
Arabinose
Cellobiose
Galactose
Glucose
Lactose
Mannose
Xylobiose
Xylose
0
1
2
3
