# Supplementary material for: Metagenomics reveals functional synergy and novel polysaccharide utilization loci in the Castor canadensis fecal microbiome
Source: ISME J. 2018 Jul 16;12(11):2757–69. doi: 10.1038/s41396-018-0215-9 (PMC6193987; doi:10.1038/s41396-018-0215-9)
Supplement: Supplementary file 8 — Supplemental Figure Captions [file 41396_2018_215_MOESM8_ESM.pdf]

Metagenomics Reveals Functional Synergy and Novel Polysaccharide Utilization Loci in the *Castor canadensis* Fecal Microbiome

Zachary Armstrong, Keith Mewis, Feng Liu, Connor Morgan-Lang, Melanie Scofield, Evan Durno, Hong Ming Chen, Kevin Mehr, Stephen G. Withers, Steven J. Hallam

Supplemental Figures

Supplementary Figure 1. Comparison of beaver fecal metagenome with other sequenced mammal microbiomes. Heatmap shows enrichment (blue) or depletion (red) of all families of CAZymes for each mammal. Clustering of mammals shows CAZyme abundance correlates with host digestive strategy. Clusters of genes enriched in herbivores include: **1)** families active on plant polysaccharides including cellulose, hemicellulose and pectin; **2)** families active on xylan. Clusters of genes enriched in carnivores include: **3)** families active on animal polysaccharides such as glycosaminoglycans.

Supplementary Figure 2. Substrates used in multiplex screening. 6-Chloro-4-methylumbelliferyl cellobioside, 6-chloro-4-methylumbelliferyl xyloside and 6-chloro-4-methylumbelliferyl xylobioside were used for functional screening. Hydrolysis of these substrates liberates 6-chloro-4-methylumbelliferone which can be detected through fluorescence spectroscopy.

Supplementary Figure 3. Deconvolution of hits. Multiplex screening of the beaver metagenomic library identified 52 clones capable of degrading one of the three substrates used. Dotted lines represent six standard deviations above the mean for each substrate used. Shown here are the results from deconvolution of 103 putative positive clones identified in the initial screen.

Metagenomics Reveals Functional Synergy and Novel Polysaccharide Utilization Loci in the *Castor canadensis* Fecal Microbiome

Zachary Armstrong, Keith Mewis, Feng Liu, Connor Morgan-Lang, Melanie Scofield, Evan Durno, Hong Ming Chen, Kevin Mehr, Stephen G. Withers, Steven J. Hallam

Supplementary Figure 4. Principal component analysis of specific activity data. The relative activity of each fosmid on nine different glycosides is projected in two dimensional space. Principal components 1 and 2 explain 45.73% and 19.16% of the variance respectively. Panel (A) shows fosmids colored according to the optimal substrate for each fosmid. Panel (B) is colored by the presence of either genes while panel (C) is colored by the presence of GH43 genes. Arrows indicate unit activity for each glycoside projected onto the same two dimensional space.

Supplementary Figure 5. Fosmids identified from high throughput screening A) Schematic representing the identified fosmids, gene presence and similarity. Grey bars represent each fosmid and are proportional to their length. Fosmids sharing 100% identity with another fosmid were removed as duplicates. Connections in the center represent areas of 90% or greater nucleotide identity between fosmids as identified by BLASTN. Inner track represents coverage of metagenomic reads mapping to fosmids. Outer coloured track represents fosmid phylogeny as determined by LCAstar. Coloured bars within each fosmid represent GH domains as predicted by BLASTP against the CAZy database**.**

Supplementary Figure 6. Gene organization of multi-domain proteins identified on functional fosmids. Proteins containing more than one domain with a CAZy annotation are shown.

Metagenomics Reveals Functional Synergy and Novel Polysaccharide Utilization Loci in the *Castor canadensis* Fecal Microbiome

Zachary Armstrong, Keith Mewis, Feng Liu, Connor Morgan-Lang, Melanie Scofield, Evan Durno, Hong Ming Chen, Kevin Mehr, Stephen G. Withers, Steven J. Hallam

Supplemental Tables

Supplementary Table 1. SSU rRNA gene pyrotag OTU counts

Supplementary Table 2. Mammalian fecal microbiome CAZyme counts

Supplementary Table 3. KEGG pathway counts for the assembled beaver fecal microbiome

Supplementary Table 4. Beaver fecal microbiome binned contig assignments and statistics

Supplementary Table 5. Beaver fecal microbiome binned contig normalized lignocellulose active CAZyme counts

Supplementary Table 6. Fosmid annotations and statistics

Supplementary Table 7. Mammalian metagenomic GH43 subfamilies

Supplementary Table 8. Activity of purified GH43 enzymes on aryl glycosides
